# Neuroanatomical Basis of State-Dependent Activity of Upper Airway Muscles

**DOI:** 10.3389/fneur.2018.00752

**Published:** 2018-09-10

**Authors:** Irma Rukhadze, Victor B. Fenik

**Affiliations:** ^1^VA West Los Angeles Medical Center, West Los Angeles, CA, United States; ^2^David Geffen School of Medicine, University of California Los Angeles, Los Angeles, CA, United States; ^3^Websciences International, Los Angeles, CA, United States

**Keywords:** obstructive sleep apnea, hypoglossal motoneurons, neurotransmitters, genioglossus, upper airway

## Abstract

Obstructive Sleep Apnea (OSA) is a common sleep-related respiratory disorder that is associated with cognitive, cardiovascular, and metabolic morbidities. The major cause of OSA is the sleep-related reduction of upper airway muscle tone that leads to airway obstructions in individuals with anatomically narrow upper airway. This reduction is mainly due to the suppressant effect of sleep on hypoglossal motoneurons that innervate upper airway muscles. The hypoglossal motoneurons have state-dependent activity, which is decreased during the transition from wakefulness to non-rapid eye movement sleep and is further suppressed during rapid eye movement sleep. Multiple neurotransmitters and their receptors have been implicated in the control of hypoglossal motoneuron activity across the sleep-wake states. However, to date, the results of the rigorous testing show that withdrawal of noradrenergic excitation and cholinergic inhibition essentially contribute to the depression of hypoglossal motoneuron activity during sleep. The present review will focus on origins of noradrenergic and cholinergic innervation of hypoglossal motoneurons and the functional role of these neurons in the state-dependent activity of hypoglossal motoneurons.

## Neurotransmitters implicated in the control of hypoglossal motoneurons

Obstructive Sleep Apnea (OSA) is a sleep-related breathing disorder characterized by repetitive nocturnal apnea/hypopnea episodes due to partial or complete closure of upper airway (1–5). The resulting chronic intermittent hypoxia, hypercapnia, and frequent arousals that are accompanied by sympathetic and cardiovascular activations lead to sleep fragmentation and cognitive impairments, as well as cardiovascular and metabolic morbidities (3, 6–11).

In most OSA patients, the nocturnal apneic episodes result from anatomical abnormalities of upper airway aperture that are combined with the sleep-related depression of upper airway muscle tone (3, 5). Hypoglossal motoneurons innervate upper airway muscles including the genioglossus muscle, the main tongue protruder muscle, which plays the critical role in maintaining upper airway patency (12–17). The elevated activity of upper airway muscles, including the genioglossus muscle, keeps the airway open during wakefulness in OSA patients (18). However, their activity is reduced during non-rapid eye movement (NREM) sleep and further suppressed during rapid eye movement (REM) sleep (2, 13, 16, 18–23). Multiple neurotransmitters have been implicated in the control of state-dependent activity of hypoglossal motoneurons [reviewed by (24–27)].

The glycinergic nature of the inhibition of hypoglossal motoneuron activity during REM sleep was hypothesized based on the findings that strychnine, a glycine receptor antagonist, abolished large postsynaptic hyperpolarizing potentials that appeared in REM sleep during intracellular recording of hypoglossal motoneurons (28). However, the causal relationship between these potentials and the membrane hyperpolarization or the increase in rheobase, which are the main indicators of decreased neuronal excitability, has not been demonstrated. The involvement of glycinergic inhibition in REM sleep-related depression of hypoglossal motoneuron was also suggested by the increase of glycine release in the hypoglossal nucleus that has been detected using the microdialysis technique (29). In addition, an increase in concentration of another widespread inhibitory neurotransmitter in the central nervous system, gamma-Aminobutyric acid (GABA), was detected in these experiments (29). However, contrary to the effect of strychnine that abolished both membrane hyperpolarization and the rheobase increase in spinal motoneurons during REM sleep-induced atonia of postural muscles (30, 31), GABA or glycinergic receptor antagonists applied on hypoglossal motoneurons did not restore the activity of hypoglossal nerve during REM sleep-like state induced by injections of carbachol, a dual cholinergic agonist, into dorsolateral pontine tegmentum in decerebrated cats (32) and anesthetized rats (33). In addition, these antagonists were not effective within the hypoglossal nucleus during natural REM sleep in freely behaving rats (34). These studies provided the evidence that either GABA or glycinergic inhibition at the level of hypoglossal motor nucleus have minimal or no effect on depression of upper airway muscles during REM sleep [reviewed by (26, 27, 35, 36)].

The disfacilitatory serotonergic mechanism has been proposed to play a key role in REM sleep-related depression of hypoglossal motoneuron activity (32, 37, 38). This hypothesis was based on the findings that medullary serotonergic neurons project to hypoglossal motoneurons (39), serotonin has the excitatory effect on hypoglossal motoneurons (40), excitatory serotonergic 5HT2A receptors are expressed in the hypoglossal nucleus (41, 42); serotonergic neurons are silent during REM sleep (43) and serotonin concentration is decreased during REM sleep-like state in decerebrated cats (44) and natural REM sleep in behaving cats (45). This hypothesis was also tested by microinjections of a broad-spectrum serotonergic antagonist, methysergide, into hypoglossal motor nucleus during REM sleep-like state (46). However, despite many synergic findings supporting this hypothesis, the follow-up functional studies conducted in anesthetized and naturally sleeping rats showed that serotonin contributes minimally to REM sleep-related depression of hypoglossal motoneuron activity (47–49). In these studies, combined microinjections of methysergide and prazosin, an alpha-1 adrenergic receptor antagonist, into the hypoglossal motor nucleus in anesthetized rats abolished REM sleep-related depression of hypoglossal motoneurons (47). However separated injections of these antagonists revealed that the inhibition of noradrenergic transmission on hypoglossal motoneurons had a major contribution to the hypoglossal depression as compared to serotonergic mechanisms (47); this contribution was estimated approximately at 90% of total effect of the antagonists (26). Comparable results were obtained in naturally sleeping rats, in which the application of terazosin, an alpha-1 adrenergic receptor antagonist, into the hypoglossal motor nucleus using the reverse microdialysis technique decreased REM sleep-related suppression of respiratory activity of genioglossus muscle by ~50% (50). However, the application of serotonergic antagonists had no effect in the same preparation (48). In addition, the inhibition of serotonergic medullary raphe cells in behaving rats had minimal effects on GG activity during sleep and wakefulness (49). Furthermore, most of the brainstem noradrenergic neurons have state-dependent activity, i.e., they have highest activity during wakefulness, their firing rate is reduced in NREM sleep and it is minimal during REM sleep (51–53). Noradrenergic neurons also innervate hypoglossal motoneurons (54–56). Thus, noradrenergic system plays a critical role in suppression of hypoglossal motoneurons during REM sleep [reviewed by (24, 26, 27, 57)].

The withdrawal of glutamatergic drive has been hypothesized to contribute to REM sleep-related suppression of hypoglossal motoneurons (29, 58, 59). In support of this hypothesis, glutamatergic neurons of intermediate reticular region (IRt) of the medulla and Kolliker-Fuse nucleus send axonal projections to the hypoglossal motoneurons (60, 61). In *in vitro* studies, the transmission of glutamate to hypoglossal motoneurons was found to be pre-synaptically inhibited by muscarinic mechanisms, which may provide the state-dependent modulation of glutamatergic release in behaving animals (62). Also, the respiratory modulation of hypoglossal motoneurons is mediated by glutamatergic neurotransmission (58, 63). However, the functional role of glutamatergic transmission in the state-dependent activity of genioglossus muscle did not receive adequate support in behaving rats (59).

The effect of orexin was studied using decerebrated cats and anesthetized rats (64, 65). In both studies orexin increased genioglossus muscle activity; this increase was abolished by combined antagonism of orexin-1 and orexin-2 receptors (65). However, it is not clear whether orexinergic transmission within the hypoglossal motor nucleus is involved in depression of hypoglossal motoneurons during NREM or REM sleep.

The application of histamine into hypoglossal motor nucleus elicits powerful activation of genioglossus muscle through histamine-1 receptors in behaving rats (66). However, antagonists of histamine-1 receptors applied into hypoglossal motor nucleus did not significantly alter spontaneous genioglossus muscle activity indicating that there is little or no endogenous histaminergic excitation of hypoglossal motoneurons (66).

Cholinergic mechanisms were found to essentially contribute to the state-dependent activity of hypoglossal motoneurons (67). The application of scopolamine, a muscarinic antagonist, into the hypoglossal nucleus in behaving rats revealed that the cholinergic inhibition plays a significant role in the regulation of state-dependent activity of the genioglossus muscle during natural sleep-wake states (67). The G-protein-coupled inwardly rectifying potassium channels that are expressed in hypoglossal motoneurons have been suggested to mediate this effect (67).

**Figure 1:**
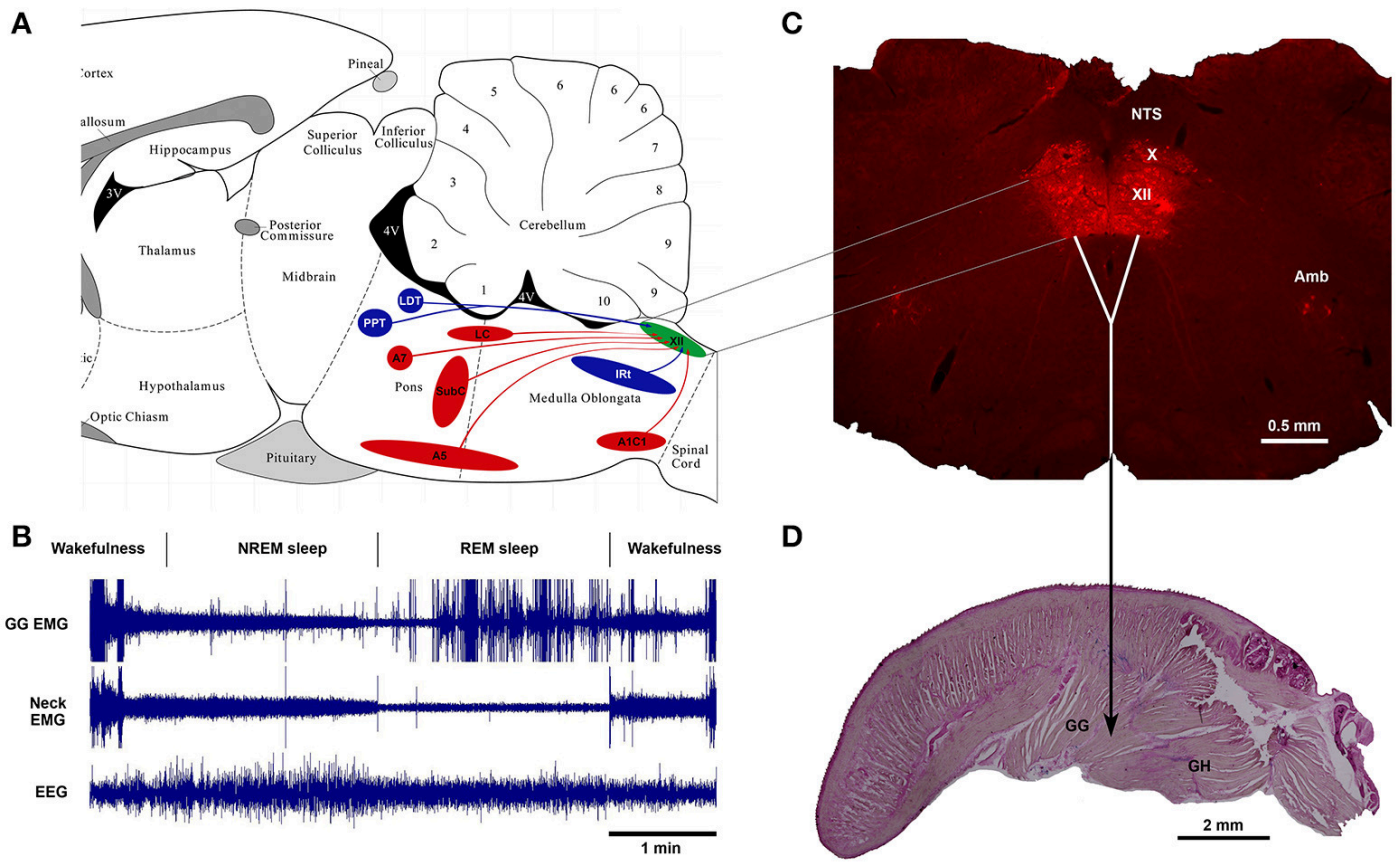
Schematics of brainstem noradrenergic and cholinergic neurons projecting to hypoglossal motoneurons that innervate genioglossus (GG) muscle and are involved in state-dependent activity of GG muscle. **(A)** the location of brainstem noradrenergic neurons and cholinergic neurons that project to the hypoglossal nucleus shown on sagittal representation of a rodent brainstem. **(B)** An example of state-dependent activity of GG muscle during wakefulness, NREM sleep and REM sleep in a naturally sleeping mouse. The GG muscle activity is markedly decreased during transition from wakefulness to NREM sleep and further reduced during REM sleep. During later period of the REM sleep, the GG muscle generates intense twitches with gradually increasing intensity toward the end of the state. **(C)** A coronal medullary section of a rat brain showing the Choline Acetyltransferase-stained motoneurons in the hypoglossal motor nucleus (XII), the dorsal motor nucleus of the vagus (X) and the nucleus of ambiguus (Amb). Sagittal section of a mouse tongue stained with the Neutral Red shows the geniohyoid (GH) muscle that forms the ventral floor for the genioglossus (GG) muscle, the major tongue protruder.

Thus, as of today, one of the major advances in sleep and respiratory neurobiology was the discovery of powerful noradrenergic and cholinergic mechanisms that are responsible for state-dependent control of upper airway muscles (47, 50, 67). The present review focuses on the sources of these two neurochemically distinct mechanisms and their functional role in sleep-related depression of upper airway muscles. Figure 1 shows schematically the main anatomical projections from catecholaminergic and cholinergic nuclei of the brainstem to the hypoglossal nucleus that innervates the genioglossus and other tongue muscles. The typical state-dependent pattern of the spontaneous activity of the genioglossus muscle is shown in the representative polygraph recording obtained in behaving mice during sleep and wakefulness (Figure 1B).

### Noradrenergic inputs to hypoglossal motoneurons

The anatomical connections between the noradrenergic neurons and hypoglossal motoneurons that innervate the genioglossus muscle were first investigated by Aldes et al. (54). This study used the retrograde tracer, peroxidase-conjugated wheat germ agglutinin, which was micro-iontophoretically injected into the hypoglossal motor nucleus of the rat. The main findings of this study were that (1) noradrenergic projections to hypoglossal motoneurons originate from pontine sub-coeruleus (SubC), A7 and A5 noradrenergic neurons; and (2) noradrenergic neurons of locus coeruleus (LC) nucleus were not retrogradely labeled from the hypoglossal nucleus. Our studies have confirmed these findings but in addition to the SubC, A5, and A7 neurons, we also found that many catecholaminergic A1/C1 neurons and scarce LC neurons send axonal projections to the hypoglossal motor nucleus (55, 56).

To identify if A1/C1 neurons innervate genioglossus motoneurons, we injected a cre-dependent anterograde tracer (EF1a-FLEX-hChR2(H134R)-eYFP-AAV10) into the A1/C1 region in tyrosine hydroxylase (TH)-cre mice. We found that TH-positive anterogradely labeled axon terminals from the A1/C1 region were mainly distributed in the ventral sub-division of hypoglossal motor nucleus (56) where genioglossus motoneurons are located (15, 68–70).

The activity of noradrenergic LC and SubC neurons changes across sleep-wake states; their firing rate is highest during wakefulness, reduced at the onset of NREM sleep and minimal or abolished during REM sleep (51–53). Recently, we found a significant correlation between cFos expression in noradrenergic neurons of SubC, A5, A7, and A2/C2, but not A1/C1, and the amount of time spent in pharmacologically induced REM sleep-like state. This suggests that, similar to LC and SubC neurons, the A5, A7, and A2/C2 neurons also have state-dependent activity whereas the activity of the ventrolateral medullary A1/C1 neurons is not changed with the vigilant states (53). The state-dependent pattern of activity of most brainstem noradrenergic neurons projecting to hypoglossal motoneurons, prompted to hypothesize that these neurons may contribute to the REM sleep-related suppression of upper airway muscles by withdrawal of excitatory noradrenergic drive to hypoglossal motoneurons during REM sleep [reviewed by (38)].

In functional studies, the role of endogenous noradrenaline and serotonin was tested by using antagonists of noradrenergic and serotonergic receptors applied at the hypoglossal motoneuronal pool in anesthetized and behaving rats (47, 48, 50). In support of the disfacilitation hypothesis the latter studies provided the evidence that the withdrawal of mainly noradrenergic and, to a lesser extent, serotonergic drives to upper airway motoneurons during REM sleep is the major cause of REM sleep-related depression of hypoglossal motoneurons in anesthetized rats (47). Comparable results were obtained for noradrenergic mechanisms in behaving rats (50). However, the serotonergic effects were not significant in behaving rats [(48); reviewed by (24, 26, 27)]. Based on additional analysis of the antagonist effects in anesthetized rats, a neuronal network was proposed, in which the noradrenergic and serotonergic drives to hypoglossal motoneurons are mediated via additional excitatory and inhibitory interneurons, respectively (26). The main basis for the proposed network was the concept that the adrenergic and serotonergic antagonists injected into the hypoglossal nucleus diffused outside the nucleus and block corresponding receptors leading to the abolition of the hypoglossal motoneuron depression during REM sleep-like state (71). This diffusion hypothesis has received an experimental support in our preliminary studies suggesting that the noradrenergic drive to the hypoglossal nucleus is not direct (72). In addition, our recent collaborative computational study validated this network and revealed the dynamics of interaction between the monoaminergic neurons and both excitatory and inhibitory interneurons during NREM and REM sleep (73).

The important question regarding which noradrenergic neurons mostly affect hypoglossal nerve activity was studied in urethane-anesthetized rats. We tested the effect of pharmacological inhibition of noradrenergic A7, SubC, LC, and A5 groups on the level of hypoglossal nerve activity and found that the inhibition of A7 neurons significantly decreased the hypoglossal nerve activity whereas the inhibition of A5, LC, or SubC neurons did not have any effect (74–76). This data suggested that the A7 neurons provide the major NA excitatory drive to hypoglossal motoneurons among the tested noradrenergic groups (75).

The involvement of A1/C1 catecholaminergic neurons in the control of the activity of hypoglossal motoneurons was recently studied using a chemogenetic technique. Since the A1/C1 neurons have been suggested to have activity that is not dependent on the vigilant states (53), they could be involved in non-state-dependent control of hypoglossal motoneurons. However theoretically, there is a possibility that the release of noradrenaline from A1 terminals within the hypoglossal nucleus is modulated through some sleep-specific presynaptic inhibitory mechanisms, similar to the discovered earlier, presynaptic cholinergic control of glutamate release to hypoglossal motoneurons (62). To this end, we recently tested the role of medullary A1/C1 neurons in control of the activity of genioglossus muscle using the “designer receptor exclusively activated by a designer drug” (DREADD) technique (77). A Cre-dependent viral vector hSyn-Dio-hM4Di-mCherry-AAV10 was microinjected into the A1/C1 region, which resulted in the expression of inhibitory receptors in the A1/C1 neurons in behaving dopamine β-hydroxylase (DBH)-cre mice, in which the Cre-recombinase is expressed in all catecholaminergic neurons. Following the expression of hM4Di in A1/C1 neurons, systemic injections of the clozapine-N-Oxide (CNO) inhibited A1/C1 neurons that resulted in decreased activity of genioglossus muscle. This suggested that A1/C1 neurons provide a net excitatory effect on the activity of upper airway muscles. However, the relative effect of CNO on the genioglossus activity was similar during both wakefulness and NREM sleep suggesting that A1/C1 neurons do not contribute to depression of genioglossus activity during transition from wakefulness to NREM sleep (77).

## Cholinergic inputs to hypoglossal motoneurons

The anatomical connections between cholinergic neurons and hypoglossal motoneurons were first investigated by Woolf and Butcher (78) using fluorescent retrograde tracers that were iontophoretically applied into the hypoglossal motor nucleus in rats. This study reported that cholinergic innervation of hypoglossal motoneurons originates from pontine laterodorsal (LDT) and pedunculopontine (PPT) tegmental nuclei. The contribution of PPT neurons to this innervation was larger than those from LDT and the projections were mainly ipsilateral (78).

In our studies, we injected retrograde tracers, FluoroGold and Cholera toxin B subunit, into the hypoglossal motor nucleus by an air pressure-driven delivery system (79, 80). In agreement with the earlier study of Woolf and Butcher (78), ~1% of PPT/LDT cholinergic neurons projected to the hypoglossal motor nucleus. However, the PPT/LDT projections to hypoglossal motoneurons were bilateral (79). In another study, we found that ~40% of cholinergic neurons of the caudal IRt region in medulla projected to the hypoglossal nucleus (80). These findings suggest that the largest cholinergic input to the hypoglossal motoneurons originates from the caudal medullary IRt region. We also found that cholinergic neurons that innervate hypoglossal motoneurons express mRNA for both muscarinic and nicotinic receptors, with the significantly high percentage of M2 muscarinic receptors present in cholinergic neurons retrogradely labeled from hypoglossal motor nucleus (80). This data suggests that the cholinergic projections from the caudal IRt region is the principal source of cholinergic drive to hypoglossal motoneurons.

Since many PPT/LDT cholinergic neurons have state-dependent activity across sleep-wake states, i.e., more active during REM sleep or wakefulness, or both, as compared to NREM sleep (81–89), the cholinergic PPT/LDT neurons that project to the hypoglossal nucleus may contribute to pre- or post-synaptic inhibition of hypoglossal motoneurons during REM sleep. The cholinergic neurons of IRt located in caudal medullary region have also been suggested to have state-dependent activity (90). This would implicate them in the sleep-related control of hypoglossal motoneurons. However, the recording of the activity of cholinergic IRt neurons during sleep-wake states is needed to confirm their role in the mechanisms of hypoglossal motoneuron suppression during NREM sleep and/or REM sleep.

Cholinergic effects on hypoglossal motoneurons are mediated through nicotinic and muscarinic receptors (67, 91). The α_3_, α_4_, α_7_, and β_2_ sub-units of nicotinic receptors (92–98) and muscarinic M1, M2, M3, M4, and M5 receptors, with the predominance of M2 receptors, are expressed in hypoglossal motoneurons (80, 99–102).

*In vitro* studies showed that the application of nicotinic receptor agonists excites hypoglossal motoneurons in neonatal rat (96). Also, the muscarinic receptors mediate a presynaptic cholinergic inhibition of excitatory glutamatergic transmission to hypoglossal motoneurons *in vitro* (62). In adult anesthetized rats, the activation of nicotinic and muscarinic receptors has respectively excitatory and inhibitory effects on the activity of genioglossus muscle (91).

The recent elegant study performed by Grace et al. (67) showed that a broad-spectrum muscarinic receptor antagonist, scopolamine, applied into the hypoglossal motor nucleus via reverse microdialysis technique significantly increased activity of genioglossus muscle during wake, NREM, and REM sleep. This study provided a strong evidence that cholinergic transmission mediated by the muscarinic receptors importantly contributes to the suppression of genioglossus muscle activity during both NREM sleep and REM sleep (67).

## Conclusion

The significant advances have been made over last three decades in our understanding of the neurochemical mechanisms that mediate the depression of upper airway muscles during NREM sleep and further suppression in REM sleep. The powerful noradrenergic and cholinergic mechanisms with minor contribution of serotonergic drive have been shown to be responsible for state-dependent control of upper airway muscles. However, the key neural groups contributing to these mechanisms have not been yet identified. The studies summarized in the present review provides the strong anatomical and physiological foundation for future basic and translational studies, which are instrumental to obtain a comprehensive knowledge of neural circuitry underlying the OSA pathophysiology and may help to define new therapeutic targets for OSA treatment.

## Author contributions

IR and VF equally contributed to writing and editing the manuscript.

### Conflict of interest statement

The authors declare that the research was conducted in the absence of any commercial or financial relationships that could be construed as a potential conflict of interest.
